# Research on emergency truck dispatching scheme for the high speed railway freight interruption

**DOI:** 10.1371/journal.pone.0346970

**Published:** 2026-04-28

**Authors:** Shuaixin Guo, Zhuojun Hu, Su Zhao, Jia Feng

**Affiliations:** 1 School of Economics and Management, Beijing Jiaotong University, Beijing, China; 2 Henan Railway Construction Investment Group Co., Ltd., Zhengzhou, Henan, China; 3 School of Traffic and Transportation, Beijing Jiaotong University, Beijing, China; 4 Integrated Transport Research Center of China, Beijing, China; 5 Key Laboratory of Transport Industry of Big Data Application Technologies for Comprehensive Transport, Beijing Jiaotong University, Beijing, China; The Hong Kong Polytechnic University, HONG KONG

## Abstract

Subject to the risk of disruptions in high-speed rail (HSR) logistics networks caused by natural disasters or equipment failures, this study proposes an emergency scheduling optimization framework based on truck transshipment. By establishing a Mixed-Integer Linear Programming (MILP) model that integrates vehicle deployment point selection, route planning, and timeliness constraints, it achieves, for the first time, multi-level collaborative decision-making covering “vehicle deployment point selection - truck scheduling - goods transshipment” following an HSR logistics disruption. An Adaptive Large Neighborhood Search (ALNS) algorithm is designed, incorporating a dynamic strategy combining destroy operators (random/worst/Shaw/depot consolidation removal) and repair operators (greedy/regret-2/regret-3 insertion) to generate high-quality scheduling schemes. Using both the Zhengzhou-Qingdao Express Rail Line disruption case and multi-scale random instances, the model’s effectiveness is validated: ALNS achieves solution quality comparable to CPLEX with a maximum gap of only 0.037% while substantially reducing computation time, and significantly outperforms GA in both solution quality and efficiency.

## 1. Introduction

High-speed rail (HSR) logistics, leveraging its inherent efficiency and punctuality, is increasingly becoming a critical backbone of modern supply chains. However, as HSR logistics systems operate on HSR transportation networks, they inevitably face the risk of line disruptions caused by unexpected events such as natural disasters, equipment failures, and human-induced accidents. These disruptions can trigger a chain reaction including cargo backlogs, delivery delays, and cascading supply chain failures, thereby threatening supply chain stability and economic security [1].

**Fig 1:**
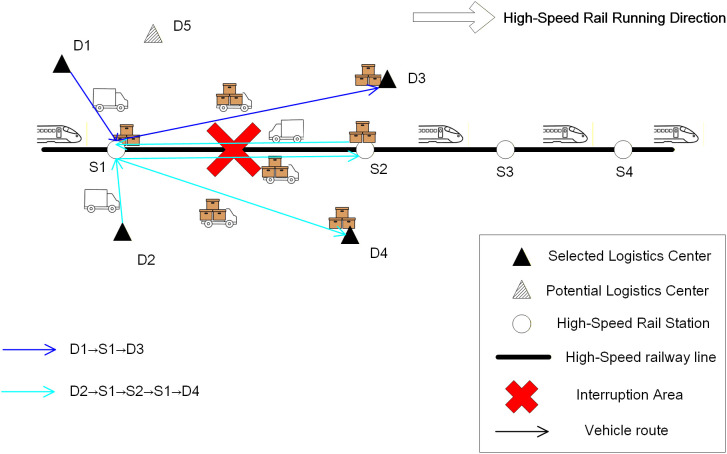
HSR logistics network with disruption and rerouting strategies.

This study addresses scenarios involving disruptions on HSR lines used for logistics operations, proposing an emergency dispatching solution that utilizes freight trucks for transshipment. Specifically, by selecting logistics centers as deployment points, freight trucks are dispatched from these deployment points to directly transship goods destined for the immediate next station to the corresponding logistics center via roadway, while simultaneously transshipping all remaining goods (destined for stations beyond the immediate next station) to the next accessible HSR station using freight trucks. Subsequently, railway operators dispatch alternate high-speed trains to resume transportation for the latter category of goods. The entire process is shown in Fig 1.

Due to vehicle capacity constraints in this scenario, it is infeasible to satisfy all demand within a single service. Consequently, activated freight trucks may need to perform multiple trips throughout the planning horizon. This scenario is modeled as a Vehicle Routing Problem (VRP) featuring Split Deliveries – allowing destination stations to be visited multiple times. An Adaptive Large Neighborhood Search (ALNS) algorithm is designed to solve this problem.

## 2. Background

This study concentrates on the post-disruption freight truck emergency dispatching problem for HSR logistics. Nevertheless, research on post-disruption emergency dispatching for HSR logistics, as an emerging transportation mode, remains in its early stages. Therefore, this study reviews the foundational research across the following four dimensions: HSR logistics disruptions, logistics disruptions in other transportation modes, VRP with Split Delivery characteristics, research on the ALNS algorithm.

### 2.1. Research on HSR logistics disruptions

Regarding responses to HSR network disruptions under complex disaster scenarios, Tang et al. [2] further integrated collaborative optimization of train rescheduling and passenger rerouting. Employing a Rolling Horizon Algorithm to address multi-line disruptions caused by earthquakes, they validated the model’s effectiveness in reducing emergency relief delay rates within the Guangdong-Hong Kong-Macao Greater Bay Area case study. To enhance response capability under timeliness constraints, Li et al. [3] developed an HSR seismic recovery model based on timetable rescheduling, achieving network functional restoration by integrating dynamic transportation demand prediction. Hong et al. [4] constructed a spatio-temporal network model to optimize train rescheduling and passenger reassignment for large-scale HSR disruptions.These studies focus on HSR passenger disruption scenarios. The coordinated response frameworks they established provide significant theoretical reference for emergency dispatching during HSR logistics disruptions.

In terms of network structure design, Li et al. [5] proposed an “multi-tiered hub-and-spoke collection-distribution network” architecture for HSR express services, optimizing hub location and intermodal routes through a Mixed-Integer Programming (MIP) model. This research confirmed the pivotal role of proximate distribution centers in reducing transportation costs. Nevertheless, it did not address dynamic resource scheduling post-disruption, thus providing modeling reference for goods transshipment under disruption scenarios in this study.

### 2.2. Research on logistics disruptions in other transportation modes

Research on emergency dispatching in other transportation modes provides methodological support for HSR disruption management:

Multimodal Network Reconstruction Strategies: Studies on highway and maritime disruptions emphasize the importance of network resilience reconstruction. Abadi et al. [6] proposed a “resource-path reconfiguration” framework for port disruptions, maintaining system performance by activating non-economic backup paths. This concept is transferable to the design of backup networks for HSR distribution centers. In container shipping, Viljoen et al. [7] identified critical nodes in the global shipping network based on link significance, demonstrating that topological structure analysis enhances disruption response efficiency—providing insights for protecting critical HSR logistics hubs. Ahmadinejad et al. [8] developed a Mixed-Integer Programming (MIP) model to optimize path reconfiguration for rail-highway intermodal disruptions. Empirical findings substantiate that highway substitution strategies can reduce transportation costs during rail terminal disruptions. Badyal et al. [9] devised a two-stage stochastic programming model to optimize intermodal hub layout. Wang et al. [10] proposed a stochastic programming model for port-hinterland networks, introducing Conditional Value at Risk (CVaR) to quantify costs in worst-case disruption scenarios.

Real-Time Decision Support Systems: Highway freight disruption research has achieved operational online rescheduling technologies. Karam et al. [11] developed a hybrid simulation-optimization framework for trunk line freight, generating delay mitigation strategies within 6 seconds while balancing cost, reliability (delay rate), and carbon emission objectives. In rail transport, Sato et al. [12] developed a real-time locomotive rescheduling model achieving an 8-fold computational acceleration within a 72-hour recovery period. These outcomes provide an algorithmic foundation for real-time routing optimization of freight trucks during post-HSR disruption transshipment. François et al. [13] pioneered the application of ALNS to multi-trip VRP, utilizing dynamic operator combinations to address urban distribution disruptions under time window constraints, achieving a 15-fold speedup over traditional heuristics. However, their single-mode optimization approach is ill-suited to meet the resource coordination requirements inherent to multimodal transportation systems.

### 2.3. VRP with demand splitting features

The Split Delivery Vehicle Routing Problem (SDVRP) significantly enhances logistics system flexibility by relaxing the rigid constraints of the traditional Capacitated VRP (CVRP), permitting single-customer demands to be served by multiple vehicles through split deliveries. The VRP variants discussed in the following studies all incorporate this demand splitting feature and predominantly employ heuristic algorithms for solution—aligning precisely with the objectives of this research.

Hernández-Pérez et al. [14] designed a two-phase heuristic algorithm: an initial solution construction phase using Variable Neighborhood Search (VNS), followed by local optimization through a branch-and-cut algorithm, successfully solving large-scale instances with up to 500 nodes. In subsequent work, Hernández-Pérez et al. [15] proposed a branch-and-cut algorithm for exactSDVRP solutions; however, its computational time exhibits exponential growth with problem scale, rendering it impractical for real-world large-scale scenarios. Hernández-Pérez et al. [16] further developed a branch-and-cut algorithm for the Single Commodity Pickup and Delivery Problem with Split Deliveries (SDOPDP), introducing a “temporary transshipment depot” mechanism.

To overcome computational efficiency limitations, Eydi et al. [17] incorporated the Simulated Annealing (SA) algorithm into a reverse logistics SDVRP. By integrating fuel consumption optimization objectives, they achieved a 20-fold acceleration in solving speed while maintaining solution quality, validating the effectiveness of heuristics under complex constraints. Ma et al. [18] proposed a Genetic Quantum Algorithm, transforming task assignment into a Transportation Problem solution approach. Yang [19] innovatively fused Tabu Search and Simulated Annealing (TSA), designing capacity constraint repair and vehicle replacement operators. This achieved improvements in both solving speed and solution quality on the Solomon benchmark datasets. Xiong et al. [20] introduced the novel strategies “demand pre-splitting” and “dynamic splitting” under shortest path constraints, substantially enhancing solving efficiency.

### 2.4. ALNS algorithm

Numerous enhanced ALNS algorithms have been proposed in recent years to solve VRP and their variants.

Ropke and Pisinger [21] pioneered the ALNS algorithm, employing dynamic combinations of multiple destroy and repair operators to solve the Pickup and Delivery Problem with Time Windows (PDPTW). Azi et al. [22] designed hierarchical ALNS operators (customer/route/workday level) for Multi-Trip Vehicle Routing Problems (MTVRP). François et al. [13] applied ALNS to the Multi-Trip VRP with Time Windows (MTVRPTW), enhancing solution quality through integrated route and trip assignment (contrasting traditional decoupled approaches). Their integration of auto-configuration tools to optimize operator combinations provided novel insights for urban logistics scheduling. Sun et al. [23] hybridized the Biogeography-Based Optimization (BBO) algorithm with ALNS for the Location-Routing Problem with Split Deliveries (LRP-SD), dynamically adjusting operator weights using self-pickup demand prediction. Zhang et al. [24] designed a two-stage solution evaluation mechanism (segmented validation + topological sorting) for airport baggage transshipment, significantly improving ALNS feasibility verification efficiency in complex dependency scenarios.

Compared with other heuristic methods, the destroy and repair operators in ALNS are more selective and targeted rather than random. This enables more strategic reinsertion of removed nodes. Therefore, this study employs the ALNS algorithm to solve the complex multi-constrained VRP inherent in this project.

### 2.5. Positioning of this study

Addressing the research gaps identified, this study achieves, for the first time, multi-level collaborative decision-making covering “vehicle deployment point selection - freight truck scheduling - goods transshipment” following HSR logistics disruptions. Focusing specifically on post-disruption freight truck emergency dispatching, we integrate the ALNS algorithm with a vehicle deployment point selection mechanism. This establishes a multi-level collaborative decision framework balancing timeliness guarantees and economic feasibility. The empirical case study on China’s Zhengzhou-Qingdao HSR Freight Corridor validates the effectiveness of both the model and solution methodology.

## 3.. Problem description

**Fig 2:**
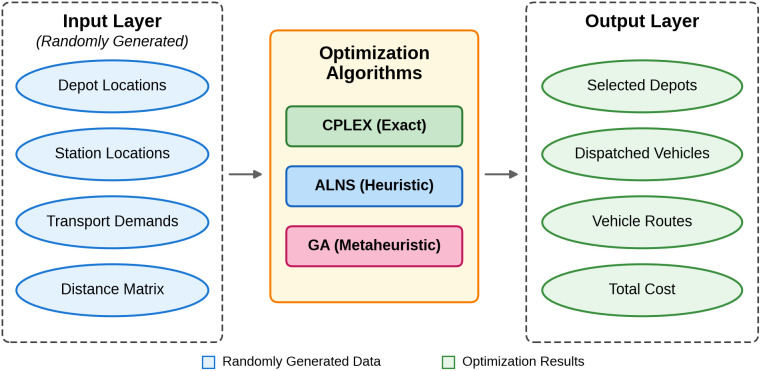
ALNS-based integrated optimization framework for emergency truck dispatch in HSR disruptions.

Within the emergency response to unidirectional disruptions in HSR freight logistics, deployment point selection and freight truck scheduling constitute the core problems. This study establishes a Mixed-Integer Linear Programming (MILP) model to rationally select deployment point for freight truck dispatch. Under the constraints of permissible delay times, it scientifically allocates freight truck resources, optimizes route planning, and achieves rapid goods transshipment while minimizing costs. ALNS-based integrated optimization framework for emergency truck dispatch in HSR disruptions is shown in Fig 2.

### 3.1 Problem assumption

After the interruption of HSR freight transportation, various uncertain factors will arise, and these changes are difficult to summarize with a single model. This article studies problems in typical contexts. Before model construction, the following assumptions are made to simplify the problem, eliminate interference from secondary factors, and facilitate model calculation:

1. Railway operators can promptly schedule high-speed trains to complete transportation tasks for stations beyond the disruption point.
2. Only static goods transshipment demand is considered.
3. The locations, distances, travel times, demand quantities, and other parameters are known and deterministic for all nodes.
4. Deployment points possess a sufficient number of vehicles (but subject to maximum dispatch quotas) in good operating condition and ready for immediate deployment.
5. No secondary disruptions occur during the transportation process.

### 3.2. Notations

The notations used in the model are shown in Table 1.

**Table 1:** Symbols and their representations.

Symbols	Representations
**Basic parameters**	
K	K={1,2,\ldots,k}, the set of candidate vehicles, k is the total number of vehicles, and each vehicle has its own deployment point.
*S*	$S=S_{d}⋃S_{u}$,the set of HSR freight stations, where $S_{d}$ denotes the subset of disrupted freight stations where goods are stranded, and $S_{u}$ denotes the subset of downstream transfer stations.
D	D={1,2,\ldots,d}, the set of candidate logistics centers, where *d* is the number of candidate logistics centers. Elements in *D* may be activated as vehicle deployment points. Both *S* and *D* may appear together in some routing-related constraints because they are both transshipment nodes; however, only elements in *D* can be selected as vehicle deployment points and dispatch vehicles.
IJ	$IJ=\left\{ij|i\in S_{d},j\in S_{u}⋃D\right\}$, the set of routes for goods to be transported.
$r_{kd}$	$r_{kd}\in \left\{0,1\right\}$, equals to one if the vehicle k∈K belongs to the deployment point d∈D; otherwise, equals to zero.
$d_{ij}$	The distance of the vehicle from node i∈S⋃D to node j∈S⋃D.
$t_{ij}$	The travel time of the vehicle from node i∈S⋃D to node j∈S⋃D.
$\overset{H}{\underset{ij}{t}}$	The normal HSR transportation time from disrupted freight station *i* ∈ $S_{d}$ to downstream transfer station *j* ∈ $S_{u}$, used as the benchmark travel time in Equation (5).
τ	Loading and unloading time per unit of cargo.
$f_{\text{1}}$	The fixed cost of selecting a logistics center as a vehicle deployment point.
$f_{\text{2}}$	Per-trip fixed dispatch cost.
$f_{\text{3}}$	Unit transportation cost per ton-kilometer.
$f_{\text{4}}$	Handling cost per unit of cargo.
$f_{\text{5}}$	Per-kilometer fuel cost.
$q_{ij}$	The total amount of goods that need to be transported from node i to node j.
C	Maximum payload capacity of vehicle.
R	Maximum number of a transfers per vehicle.
$T_{allow}$	Maximum allowable delay time.
$c_{d}$	The maximum number of vehicles that can be dispatched from the logistics center d.
**Intermediate variable**	
$\overset{\text{0}}{\underset{k}{t}}$	The dispatch time of vehicle k.
$\overset{r}{\underset{k}{t}}$	The total travel and loading/unloading time for the r-th transfer of vehicle k.
**Decision variables**	
$z_{d}$	$z_{d}\in \left\{0,1\right\}$, equals to one if logistics center d∈D is chosen as the vehicle deployment point; otherwise, equals to zero.
$\overset{kr}{\underset{ij}{x}}$	$\overset{kr}{\underset{ij}{x}}\in \left\{0,1\right\}$, equals to one if the r-th transfer of vehicle k∈K from node i to node j; otherwise, equals to zero.
$\overset{kr}{\underset{ij}{p}}$	The quantity of goods transported from node i to node j for the r-th transfer of vehicle k.
$\overset{k}{\underset{dj}{y}}$	$\overset{k}{\underset{dj}{y}}\in \left\{0,1\right\}$,equals to one if vehicle k is allocated from logistics center d to node i; otherwise, equals to zero.

### 3.3. Objective function

Equation (1) is the objective function, considering all costs of cargo transportation after interruption, including the fixed cost of selecting the logistics center as a deployment point, the fixed cost of using freight trucks, the unit transportation cost of freight trucks, the unit loading and unloading cost of goods, and the driving cost of freight trucks. Specifically, this function achieves rapid and effective utilization of transportation resources in emergency situations by minimizing the usage and dispatch costs of the deployment point.


$$minZ=\sum_{d\in D}f_{\text{1}}\cdot z_{d}+\sum_{d\in D}\sum_{k\in K}\sum_{i\in S⋃D}\left(f_{\text{2}}+f_{\text{5}}\cdot d_{di}\right)\cdot \overset{k}{\underset{di}{y}}+\sum_{i\in S⋃D}\sum_{j\in S⋃D}\sum_{k\in K}\sum_{r\in R}\left(f_{\text{3}}\cdot d_{ij}\cdot \overset{kr}{\underset{ij}{p}}+f_{\text{4}}\cdot \overset{kr}{\underset{ij}{p}}+f_{\text{5}}\cdot d_{ij}\right)\cdot \overset{kr}{\underset{ij}{x}}$$ (1)

### 3.4. Constraints

The constraints of the model are as follows.

The constraints on the selection of deployment points are shown in Equation (2)-(4). Equation (2) indicates that only logistics centers selected as deployment points can dispatch vehicles. Equation (3) indicates that only when the vehicle belongs to the deployment point can the next dispatch be completed. Equation (4) represents selecting at least one logistics center to ensure that there is at least one deployment point in the system for dispatching vehicles.


$$\overset{k}{\underset{dj}{y}}\leq z_{d},\forall d\in D,j\in S⋃D,k\in K$$ (2)

$$\overset{k}{\underset{dj}{y}}\leq r_{kd},\forall d\in D,j\in S⋃D,k\in K$$ (3)

$$\sum_{d\in D}z_{d}\geq 1$$ (4)

The constraint on total transit time is shown in Equation (5). Equation (5) represents that the total transit time $T_{max}$ of the goods is within the allowable delay time range. The total transit time of the goods is less than or equal to the sum of the maximum allowable delay time and the high-speed rail transportation time during normal transportation.


$$T_{max}\leq T_{allow}+\overset{H}{\underset{ij}{t}},\forall i\in S_{d},j\in S_{u}$$ (5)

Equations (6) to (9) describe the calculation method for the total transit time. Among them, equation (6) defines the total transit time $T_{max}$, which is the maximum value of all vehicle transit times. Equation (7) further expands the transportation time $T_{k}$ of a single vehicle, including two parts: dispatch time $\overset{\text{0}}{\underset{k}{t}}$, transportation and loading/unloading time $\overset{r}{\underset{k}{t}}$.


$$T_{max}\geq T_{k},\forall k\in K$$ (6)

$$T_{k}=\overset{\text{0}}{\underset{k}{t}}+\sum_{r}\overset{r}{\underset{k}{t}},\forall k\in K,r=1,2,\text{\textbackslash{}ldots},R$$ (7)

$$\overset{\text{0}}{\underset{k}{t}}=\sum_{d\in D}\sum_{i\in S⋃D}t_{di}\overset{k}{\underset{di}{y}},\forall k\in K$$ (8)

$$\overset{r}{\underset{k}{t}}=\sum_{i\in S⋃D}\sum_{j\in S⋃D}t_{ij}\cdot \overset{kr}{\underset{ij}{x}}+\tau \cdot \overset{kr}{\underset{ij}{p}},\forall k\in K,r=1,2,\text{\textbackslash{}ldots},R$$ (9)

The constraint on vehicle initial position is shown in Equation (10). Equation (10) indicates that the station where the vehicle k is first assigned must be the same as the starting station for transfer, ensuring that each vehicle is correctly assigned to the designated starting station before executing the transfer task. This constraint is to match the vehicle with the starting point of the transfer task, ensuring that the vehicle can start executing the transfer task from the correct position.


$$\sum_{d\in D}\overset{k}{\underset{di}{y}}\geq \sum_{j\in S⋃D,r=1}\overset{kr}{\underset{ij}{x}},\forall k\in K,i\in S⋃D$$ (10)

The constraints on transport task are shown in Equation (11)-(12). Transport task constraints refer to the fact that vehicles can only transport goods between a pair of origin destination (OD) points in each transport task. Equation (11) ensures that each vehicle can only select one pair of OD for service during each transfer task. The vehicle allocation constraint in equation (12) indicates that a vehicle can only be dispatched from one deployment point to prevent conflicts where freight trucks are dispatched to multiple stations simultaneously.


$$\sum_{i\in S⋃D}\sum_{j\in S⋃D}\overset{kr}{\underset{ij}{x}}\leq 1,\forall k\in K,r=1,2,\text{\textbackslash{}ldots},R$$ (11)

$$\sum_{d\in D}\sum_{j\in S⋃D}\overset{k}{\underset{dj}{y}}\leq 1,\forall k\in K$$ (12)

The constraint on vehicle scheduling is shown in Equation (13). Equation (13) ensures route continuity, meaning that if vehicle k starts its r+1 -th transfer from node j, then its r -th transfer must end at node j.


$$\sum_{i\in S\cup D}\overset{kr}{\underset{ij}{x}}\geq \sum_{i\in S\cup D}\overset{k,r+\text{1}}{\underset{ji}{x}},\;\forall k\in K,\;r=\text{1},\text{2},\ldots ,R-\text{1},j\in S\cup D$$ (13)

The constraints on the quantity of goods are shown in Equation (14)-(15). The quantity constraints of goods refer to the reasonable restriction and management of the transportation and distribution of goods during the transportation process. Equation (14) serves as a cargo capacity constraint to ensure that the quantity of goods transported by each vehicle in each transfer task does not exceed the maximum cargo capacity of the vehicle. This constraint ensures the feasibility of the cargo transportation plan and avoids overloading situations. Equation (15) ensures that all stranded goods can be promptly and completely transported to the destination station and various logistics centers through the constraint of transport completeness.


$$\overset{kr}{\underset{ij}{p}}\leq C\cdot \overset{kr}{\underset{ij}{x}},\forall k\in K,r=1,2,\text{\textbackslash{}ldots},R,i\in S⋃D,j\in S⋃D$$ (14)

$$\sum_{k\in K}\sum_{r=1}^{R}\overset{kr}{\underset{ij}{p}}\geq q_{ij},\forall i\in S⋃D,j\in S⋃D$$ (15)

The variable related constraints are shown in Equation (16).


$$z_{d}\in \left\{0,1\right\},\overset{kr}{\underset{ij}{x}}\in \left\{0,1\right\},\overset{k}{\underset{dj}{y}}\in \left\{0,1\right\},\forall k\in K,r=1,2,\text{\textbackslash{}ldots},R,d\in D,i\in S⋃D,j\in S⋃D$$ (16)

## 4. Methods

**Fig 3:**
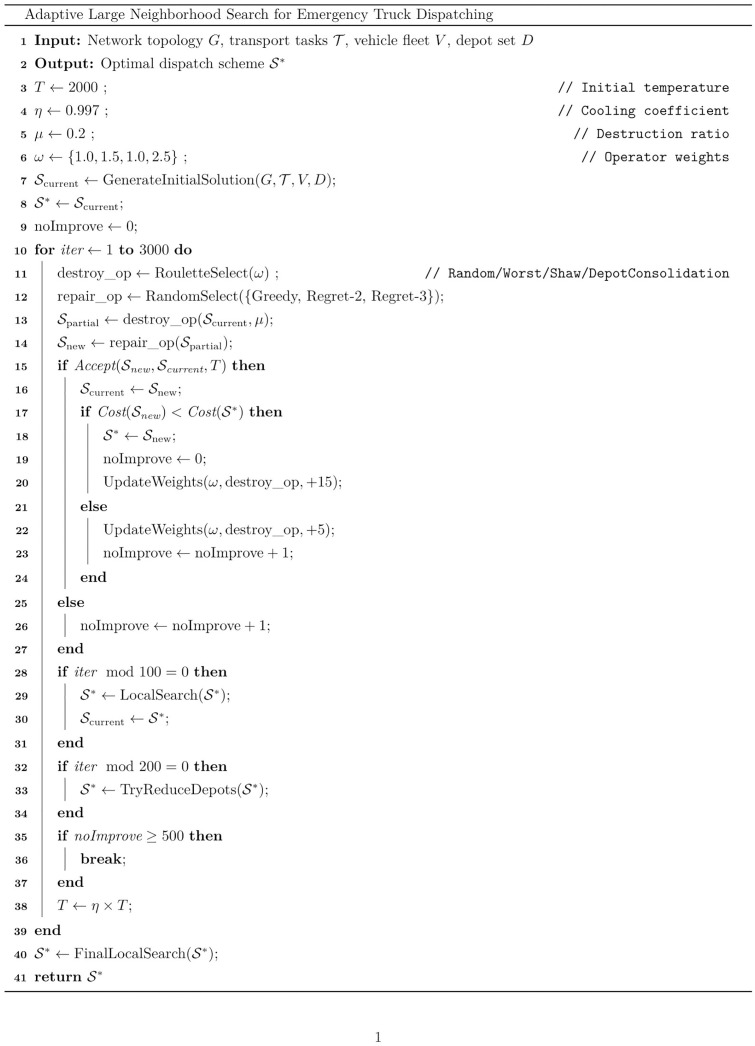
Algorithm pseudocode.

The ALNS algorithm exhibits unique advantages in addressing the strong constraints and combinatorial complexity of emergency scheduling for HSR logistics interruptions. ALNS dynamically combines selective destroy and repair operators to insert nodes into optimal positions. However, traditional heuristic methods are difficult to coordinate such complex decisions. CPLEX, as an exact solver, is limited by computational complexity and faces convergence barriers when scaling up. By adaptively adjusting the combination of operators and the strength of destruction, ALNS can solve problems while ensuring the quality of the solution. Therefore, this study adopts ALNS as the core solution framework. After initialization, the algorithm enters the main loop, continuously destroying and repairing, generating new solutions, and then deciding whether to accept the new solutions based on simulated annealing criteria. Meanwhile, the adaptive mechanism will adjust the selection probability of the operator. The temperature gradually decreases with iteration, and the algorithm eventually converges to a better solution. The algorithm pseudocode is shown in Fig 3.

As shown in the pseudocode above, the overall algorithm process is divided into three core modules:

### 4.1. Initialization phase

Upon algorithm initialization, basic parameter configuration is performed: the initial temperature parameter is set to T=2000 as the starting point for simulated annealing, allowing early acceptance of worse solutions for diversification; the destruction ratio is determined at 20%, meaning approximately 20% of tasks are removed per iteration, balancing solution diversification and reconstruction difficulty; the cooling coefficient is specified as η=0.997 for the subsequent temperature decay process, ensuring gradual exploration; the maximum number of iterations is set to 3000, sufficient for convergence while maintaining efficiency; and the no-improvement tolerance limit is established at 500 iterations as an early termination criterion. Subsequently, task preprocessing is conducted, wherein tasks are arranged in descending order by distance to prioritize long-distance assignments. Finally, a heuristic strategy is employed to generate a feasible initial solution, prioritizing the utilization of depots closest to the disrupted freight station where goods are stranded.

### 4.2. Main cycle process

The main cycle process is the core of ALNS. The algorithm core adopts an iterative optimization framework, and each iteration includes destroy and repair operations, providing a total of four removal strategies and three insertion strategies.

Destroy operation: At each iteration, one of four removal strategies is selected to systematically remove μ tasks from the current solution. The four removal strategies are as follows: the random removal strategy selects tasks randomly for removal to ensure solution diversity and escape local optima; the worst removal strategy preferentially removes tasks whose removal yields the greatest cost reduction, eliminating inefficient assignments; the Shaw removal strategy removes similar tasks with proximate destinations to exploit neighborhood coherence and enable batch reassignment; and the depot consolidation strategy removes all tasks assigned to the most wasteful vehicle deployment point, characterized by long dispatch distance relative to task volume, specifically targeting the reduction of fixed costs. [24]

Repair operation: Within the destroyed solution space, one of three insertion strategies is randomly selected to reconstruct a feasible solution. The greedy insertion strategy prioritizes long-distance tasks and inserts them at positions with minimum cost increase, providing fast reconstruction; the Regret-2 insertion strategy selects tasks with the maximum 2-regret value, that is, tasks with the largest cost difference between the optimal and second-best positions, for insertion, prioritizing tasks with higher opportunity costs; and the Regret-3 insertion strategy selects tasks with the maximum 3-regret value, considering the top three optimal positions, for insertion, which is particularly effective for problems with tight time constraints.

**Fig 4:**
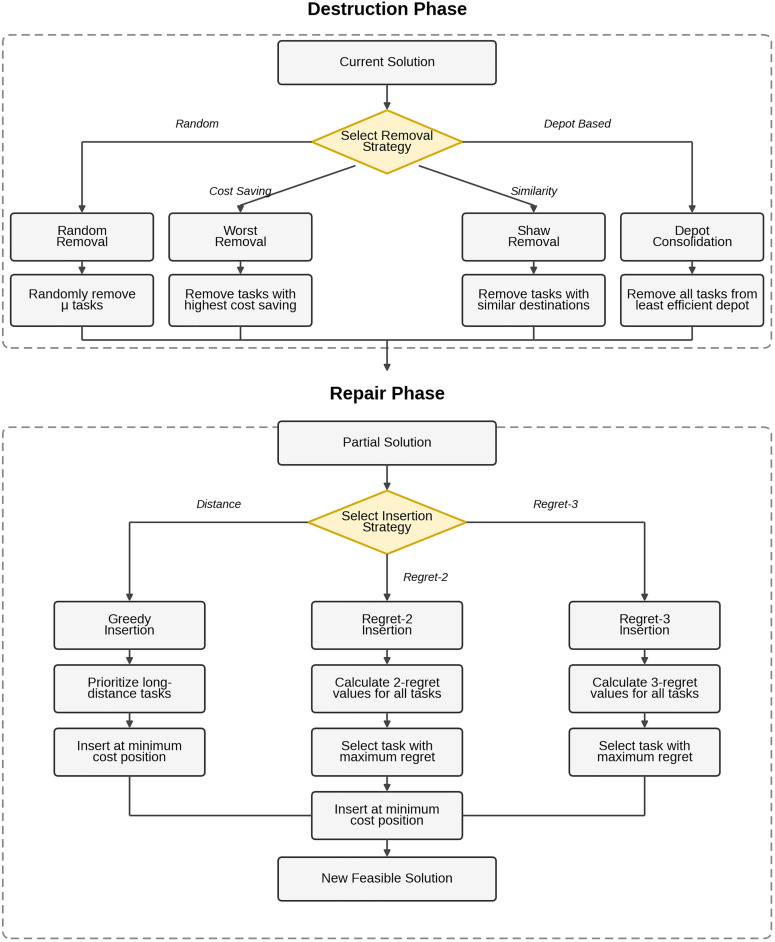
Destroy-repair procedure in Adaptive Large Neighborhood Search algorithm.

Destroy-Repair Procedure is shown in Fig 4.

### 4.3. Adaptive control mechanism

Each destruction and repair operator has a weight that is dynamically updated based on its historical performance, such as improving the quality of the solution. The algorithm employs an adaptive operator selection mechanism wherein the weights of the four destroy operators are initialized and updated every 100 iterations based on operator performance, allowing operators to accumulate meaningful statistics. The weight update formula is $\omega =max\left(0.1,0\mathrm{.8}\times \omega +0.2\times \frac{score}{count}\right)$, where finding a new best solution awards 15 points and accepting a non-improving solution awards 5 points. A simulated annealing acceptance criterion is adopted, wherein a new solution with lower cost is accepted directly, while an inferior solution is accepted with probability $exp\left(-\frac{\Delta }{T}\right)$ to escape local optima. The temperature decays according to T=η×T with η=0.997. Additionally, local search optimization is executed every 100 iterations, depot consolidation is attempted every 200 iterations to reduce fixed costs, and five rounds of final local search are performed upon algorithm termination.

This algorithm combines the characteristics of ALNS and simulated annealing. The adaptive mechanism enables the algorithm to dynamically adjust its strategy based on the performance of the operator, while the simulated annealing acceptance criterion enables the algorithm to escape local optima. By combining destroy and repair operations, the algorithm can effectively explore the solution space. The depot consolidation destroy operator is specifically designed for this problem to reduce the number of activated depots and lower fixed costs. The algorithm achieves adaptive operator selection combined with simulated annealing temperature decay, enabling intelligent balance between global exploration and local exploitation.

## 5. Case study and analysis

### 5.1. Random Instance Generation

To further validate the scalability and robustness of the proposed algorithm across different problem sizes, a set of random instances is generated to simulate various HSR freight disruption scenarios. We assume an unplanned disruption renders a section of the HSR line impassable. Station A represents the interrupted HSR freight station where goods are stranded, and Station B represents the nearest downstream transfer station. Goods stranded at Station A are handled in the following two ways: goods destined for downstream stations beyond Station B must be transshipped via roadway to Station B, then continue transportation via HSR; goods destined for local areas must be transshipped via roadway to designated logistics parks. The candidate vehicle deployment points, which also serve as logistics parks for freight delivery, are spatially distributed across the service region. Only those candidate points selected by the optimization model become activated depots.

Four scale categories are defined to represent different disruption scenarios, as shown in Table 2.

**Table 2:** Random Instance Scale Configuration.

Scale	Vehicle Deployment Points	Stations	Fleet Size	Total Demand (tons)
Small	6	8	30	120
Medium	10	12	50	200
Large	14	16	70	280
Extra-Large	20	22	100	400

Station B is located 40–80 km from Station A. Vehicles are evenly allocated across all vehicle deployment points, and the average truck speed is set to 60 km/h. The transport demand originates exclusively from Station A, with 50% destined for Station B and the remaining 50% distributed among the logistics parks according to randomly generated proportions. The baseline parameters are configured as follows: vehicle capacity C=3.5 tons, maximum allowable time $T_{allow}=240$ minutes, and Station B demand ratio = 50%. The cost parameters are specified in Table 3.

**Table 3:** Parameter settings and baseline values.

Parameter	Description	Value
$f_{\text{1}}$	Fixed establishment cost per emergency vehicle deployment point	150,000 CNY/point
$f_{\text{2}}$	Per-trip fixed dispatch cost	200 CNY/trip
$f_{\text{3}}$	Unit transportation cost per ton-kilometer	0.4 CNY/(t·km)
$f_{\text{4}}$	Loading/unloading cost per ton	30 CNY/t
$f_{\text{5}}$	Per-kilometer fuel cost (empty run)	2.5 CNY/km
τ	Loading/unloading time per ton	2 min/t
$T_{allow}$	Maximum allowable delay time	4 h
C	Freight truck maximum payload capacity	5 t

### 5.2. Multi-scale Performance Comparison

Table 4 presents the multi-scale performance comparison of CPLEX, ALNS, and GA. The computational results reveal distinct performance characteristics among the three algorithms. For the Small, Medium, and Large scale instances, CPLEX successfully obtains optimal solutions within reasonable computation times. Notably, ALNS achieves identical optimal solutions to CPLEX for the Medium and Large instances, and near-optimal solution for the Small instance with a gap of only 0.007%, demonstrating its capability to match exact optimization quality. For the Extra-Large instance, CPLEX fails to find the optimal solution within the specified time limit and only returns a feasible solution with a cost of 2,195,157. ALNS achieves the same solution quality with a cost of 2,195,157.

**Table 4:** Multi-scale performance comparison of CPLEX, ALNS, and GA.

Scale	CPLEX			ALNS		GA	
	Status	Cost	Trun(s)	Cost	Trun(s)	Cost	Trun(s)
**Small**	Opt.	626979	2.65	627025	0.87	627320	0.34
**Medium**	Opt.	943317	6.85	943317	1.31	1095351	0.41
**Large**	Opt.	1260158	7.14	1260158	5.85	1411958	0.54
**X-Large**	Feas.	2195157	–	2195157	27.02	2348796	1.77

Regarding computational efficiency, CPLEX solving time increases substantially with problem scale, from 2.65 seconds for the Small instance to 7.14 seconds for the Large instance. ALNS maintains stable computational performance across all scales, with solving times ranging from 0.87 to 27.02 seconds, achieving significant time reduction compared to CPLEX.

GA exhibits the fastest computation times, ranging from 0.34 to 1.77 seconds. However, GA consistently produces inferior solutions compared to both CPLEX and ALNS. The solution quality gap between GA and the optimal solution increases with problem scale: 0.05% for Small, 16.1% for Medium, and 12.0% for Large instances. Even for the Extra-Large instance, GA yields a solution 7.0% worse than ALNS.

In summary, ALNS demonstrates the best balance between solution quality and computational efficiency. It achieves solution quality comparable to CPLEX while substantially reducing computation time. While GA offers speed advantages, its significant solution quality degradation makes it unsuitable for practical applications where cost minimization is critical. Therefore, ALNS is recommended as the preferred algorithm for solving the emergency truck dispatching problem.

### 5.3. Real-world Case Study

**Fig 5:**
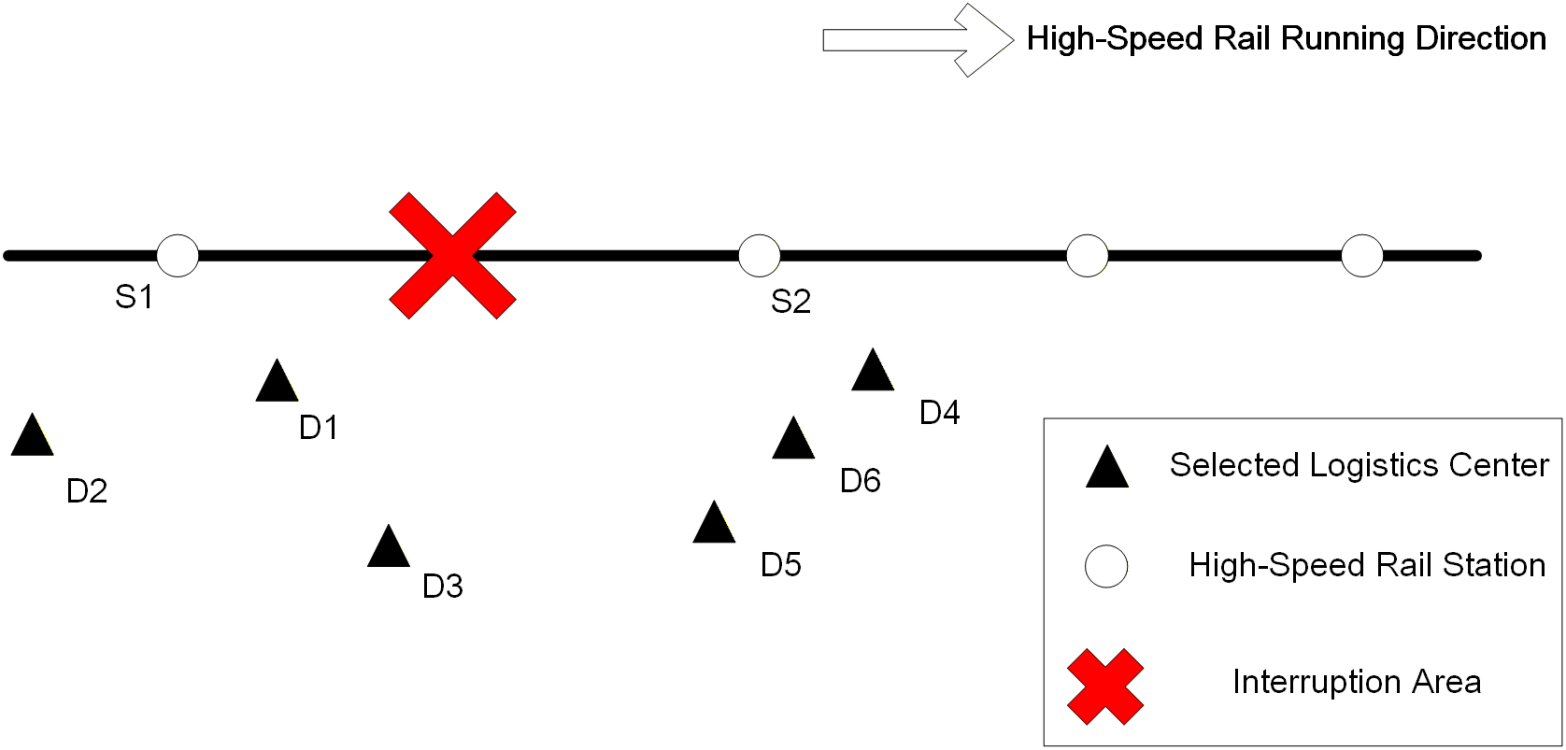
Spatial distribution of node locations.

The Zhengzhou-Qingdao HSR corridor was selected for case validation. We assume an unplanned disruption renders the section between Zhengzhou East Station (S1) and Kaifeng North Station (S2) impassable. Goods stranded at Zhengzhou East Station are handled in the following two ways. Goods destined for S2 must be transshipped via roadway to designated logistics parks in Kaifeng City; Goods destined for downstream stations beyond S2 must be transshipped via roadway to S2, then continue transportation to downstream stations via HSR. Fig 5 illustrates the geographical distribution of HSR stations and logistics centers. In the figure, D1 to D6 represent Zhengzhou Rongwan Low-Carbon Cold Chain Park, CMST Development Co., Ltd. Zhengzhou Logistics Center, Zhongyuan Logistics Park, Kaifeng Ocean Logistics Park, Mapletree (Kaifeng) Modern Logistics Park and Jixiang Logistics Park. S1 is Zhengzhou East Station. S2 is Kaifeng North Station. The distribution of node positions in the case is shown in Fig 5.

The inter-station distance between S1 and S2 is 52 kilometers, with high-speed trains normally completing the journey in approximately 30 minutes. The road distance between these stations is 60.5 kilometers, requiring about 65 minutes by road transport.

The six logistics centers D1-D6 simultaneously serve as alternative vehicle deployment points. If selected as an active deployment point, a logistics center is equipped with five freight trucks for emergency transshipment, and incurs fixed establishment costs for the deployment point. D1, D2 and D3 are located near Zhengzhou East Station. D4, D5 and D6 are located near Kaifeng North Station.

The road distances between these deployment points and the HSR stations are detailed in Table 5. Freight truck travel times between all nodes, calculated based on evening peak traffic conditions (17:00–19:00), are provided in Table 6. Travel times are derived from Baidu Maps Navigation API data capturing actual traffic conditions during the evening peak period. Given that most logistics parks are located in suburban areas with high accessibility to arterial roads, the benchmark travel times represent the verified average of repeated sampling.

**Table 5:** Distance between nodes (km).

	D1	D2	D3	D4	D5	D6	S1	S2
**D1**	0	7	10.7	62.4	54.9	60.8	12.4	61
**D2**	7	0	23.9	65.2	57.7	63.7	5.3	58.1
**D3**	11.4	23.7	0	48	40.5	46.5	28.6	52
**D4**	62.1	65.1	49.6	0	9.9	3.6	70	14.6
**D5**	54.9	58	41.7	9.7	0	6.1	62.8	11.5
**D6**	59.9	62.9	46.7	3.6	6.2	0	67.8	11.9
**S1**	14.9	6.3	30.8	71.4	63.9	69.8	0	60.5
**S2**	61.6	62	52.6	14.7	12.2	12.5	60.1	0

**Table 6:** Required freight truck travel time between nodes (min).

	D1	D2	D3	D4	D5	D6	S1	S2
**D1**	0	17	23	54	48	49	19	55
**D2**	15	0	28	57	51	53	10	53
**D3**	23	33	0	43	36	40	33	56
**D4**	56	67	47	0	19	9	62	30
**D5**	49	53	38	19	0	12	56	24
**D6**	50	52	40	8	12	0	57	28
**S1**	28	17	39	64	59	67	0	65
**S2**	57	56	58	34	28	28	54	0

According to data released by CRRC (China Railway Rolling Stock Corporation), freight electric multiple units (EMUs) operating at speeds of 250 km/h and 350 km/h have recently completed development. The freight EMU utilizes an 8-car formation, each car provides a payload capacity of 15 tonnes, yielding a maximum trainload capacity of 120 tonnes.

In the context of this case study, an impassable disruption has occurred on the S1 to Qingdao corridor, necessitating roadway transshipment of 120 tonnes of stranded cargo from S1 to S2 and designated logistics centers. Specifically, 60 tonnes (50% of total cargo), destined for downstream HSR stations beyond S2, require transshipment to S2 for onward HSR transportation. The remaining 60 tonnes are allocated to Kaifeng logistics centers: 20 tonnes to D4, 20 tonnes to D5, 20 tonnes to D6.

**Fig 6:**
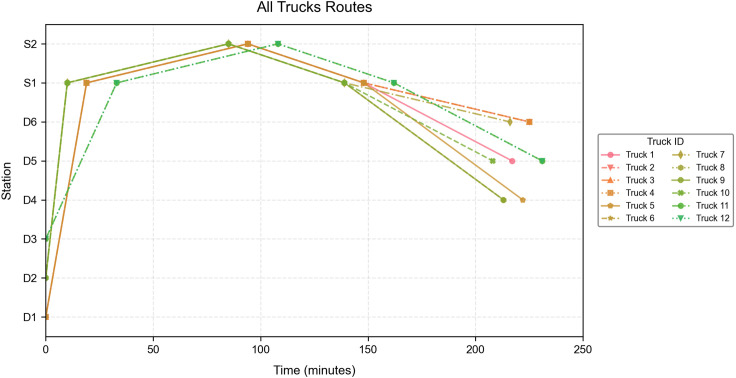
Vehicle Travel Routes.

Through modeling and solving the aforementioned case, the optimization selects logistics centers D1, D2, and D3 as active deployment points, with: D1 dispatching five freight trucks to perform the assigned transshipment missions, D2 dispatching five freight trucks, and D3 dispatching two freight trucks. The optimal objective function value reaches 471,159.3 (CNY), with a total operation duration of 231 minutes. The travel routes executed by each freight truck are illustrated in Fig 6.

Fig 6 illustrates the travel routes of the 12 freight trucks dispatched from logistics centers D1, D2, and D3. Taking the brown route in Fig 6 as an example: It represents a freight truck belonging to D1 that travels directly from D1 to S1, delivers cargo to S2, then returns to S1, and finally transships goods from S1 to D4.

The routing plan and transported cargo quantities for freight truck dispatch are detailed in Table 7.

**Table 7:** Freight truck routing plan and transported cargo quantities.

Deployment Point	Vehicle ID	Route Sequence	Freight Volume (ton)
D1	1	D1 → S1 → S2 → S1 → D5	S1 → S2: 5, S1 → D5: 5.
	2	D1 → S1 → S2 → S1 → D6	S1 → S2: 5, S1 → D6: 5.
	3	D1 → S1 → S2 → S1 → D6	S1 → S2: 5, S1 → D6: 5.
	4	D1 → S1 → S2 → S1 → D6	S1 → S2: 5, S1 → D6: 5.
	5	D1 → S1 → S2 → S1 → D4	S1 → S2: 5, S1 → D4: 5.
D2	6	D2 → S1 → S2 → S1 → D4	S1 → S2: 5, S1 → D4: 5.
	7	D2 → S1 → S2 → S1 → D6	S1 → S2: 5, S1 → D6: 5.
	8	D2 → S1 → S2 → S1 → D4	S1 → S2: 5, S1 → D4: 5.
	9	D2 → S1 → S2 → S1 → D4	S1 → S2: 5, S1 → D4: 5.
	10	D2 → S1 → S2 → S1 → D5	S1 → S2: 5, S1 → D5: 5.
D3	11	D3 → S1 → S2 → S1 → D5	S1 → S2: 5, S1 → D5: 5.
	12	D3 → S1 → S2 → S1 → D5	S1 → S2: 5, S1 → D5: 5.

## 6. Discussion

To systematically evaluate the robustness and strategic sensitivity of the HSR logistics disruption emergency dispatching model, this study conducts sensitivity analysis on three key operational parameters using the Large-scale random instance with 14 candidate vehicle deployment points and 16 stations as the baseline configuration:

(1) Freight truck maximum payload capacity. As a core indicator of vehicle resource capability, freight truck maximum payload capacity directly impacts vehicle dispatch scale and deployment point selection strategies.
(2) Maximum allowable delay time. The maximum allowable delay time reflects the intensity of timeliness constraints, determines the slack degree of time restrictions in route planning.
(3) Cargo demand distribution ratios. The distribution ratio of goods demand describes the destination distribution patterns of goods. It can be used to analyze how demand structure variations influence dispatching schemes.

### 6.1. Freight truck maximum payload capacity

The payload range for freight trucks spans 2.5 to 5 tonnes. When maximum payload capacity change, the resulting deployment point selections, number of trucks dispatched per point, and total transshipment costs are summarized in Table 8.

**Table 8:** Result summary under varying freight truck payload capacity constraints.

Freight Truck Maximum Payload Capacity (t)	Number of Vehicles Used	Number of Vehicle Deployment Points Activated	Optimal Objective Value(CNY)
2.5	50	10	1577417
3	42	9	1416123
3.5*	40	8	1260158
4	39	8	1255926
5	32	7	1097811

* denotes the baseline scenario. (The same applies below).

Analysis of the results reveals a significantly negative correlation between payload capacity and system costs. As the freight truck maximum payload capacity increases from 2.5 tonnes to 5 tonnes, the optimal objective value declines from 1,577,417 CNY to 1,097,811 CNY, a 30.4% reduction. Correspondingly, the number of vehicles used decreases from 50 to 32, while activated vehicle deployment points drop from 10 to 7.

The cost reduction pattern exhibits distinct phases. In the range from 2.5 to 3.5 tonnes, each 0.5-tonne increment yields significant savings of approximately 10–11%. However, a plateau emerges between 3.5 and 4 tonnes, where cost reduction is merely 0.3% and activated deployment points remain at 8, indicating that the system has reached a local optimum in deployment configuration.

When capacity reaches 5 tonnes, substantial improvement reoccurs with a 12.6% cost reduction and further consolidation to 7 deployment points. This suggests that the 5-tonne threshold enables structural reconfiguration of the dispatching network, allowing fewer deployment points to adequately cover total demand.

In summary, these findings—that payload capacity elevation reduces system costs—align with the core conclusion of Krammer & Schafer [25], who identified a strong negative correlation between payload capacity and unit transportation costs. This further validates the impact of vehicle resource utilization efficiency and fixed cost allocation effects on logistics system expenditures.

### 6.2. Maximum allowable delay time

Variations in the Maximum Allowable Delay Time directly influence cargo transportation urgency levels. Changes to this parameter affect: deployment point selection, number of trucks dispatched, total transshipment costs. These outcomes are summarized in Table 9.

**Table 9:** Result summary under varying maximum allowable delay time constraints.

Maximum Allowable Delay Time(min)	Number of Vehicles Used	Number of Vehicle Deployment Points Activated	Optimal Objective Value(CNY)
200	48	10	1563041
220	45	9	1411958
240*	40	8	1260158
260	33	7	1107738
280	30	6	956776

When the maximum allowable delay time increases from 200 minutes to 280 minutes, the optimal objective value decreases from 1,563,041 CNY to 956,776 CNY, representing a 38.8% reduction. Correspondingly, the number of vehicles used decreases from 48 to 30, while activated vehicle deployment points drop from 10 to 6.

The cost reduction exhibits a consistent pattern across all intervals. Each 20-minute increment yields approximately 10–14% cost savings: from 200 to 220 minutes achieves 9.7% reduction, from 220 to 240 minutes achieves 10.7%, from 240 to 260 minutes achieves 12.1%, and from 260 to 280 minutes achieves 13.6%. This increasing marginal benefit suggests that longer time allowances enable progressively more efficient network reconfiguration.

The vehicle deployment scheme demonstrates systematic consolidation as time constraints relax. Under the 200-minute constraint, the model must activate 10 deployment points to satisfy time requirements, necessitating dispatching from distant locations. As the allowable time extends to 240 minutes, the system consolidates to 8 deployment points. Further extending to 280 minutes enables complete coverage with only 6 deployment points, as the relaxed constraint allows elimination of less efficient remote dispatch locations.

Collectively, a negative correlation exists between Maximum Allowable Delay Time and the optimal objective value: As delay tolerance increases, total costs exhibit a declining trend. This aligns with Zhu et al. [26], whose study confirms that transport time window constraints directly impact intermodal solution costs, with relaxed windows substantially reducing total expenditures.

### 6.3. Cargo demand distribution

When the allocation proportion of the 120 tonnes of stranded cargo at S1 destined for S2 varies, the resulting deployment point selections, number of trucks dispatched per deployment point, and total transshipment costs are detailed in Table 10.

**Table 10:** Result summary under varying cargo demand distribution ratios.

Proportion of Cargo Destined for Station B	Number of Vehicles Used	Number of Vehicle Deployment Points Activated	Optimal Objective Value(CNY)
30%	33	7	1103526
40%	35	7	1106441
50%	40	8	1260158
60%	47	10	1564827
70%	55	11	1721080

Analysis of the results demonstrates a strong positive correlation between the proportion of cargo destined for Station B and system costs. As the proportion increases from 30% to 70%, the optimal objective value rises from 1,103,526 CNY to 1,721,080 CNY, representing a 56.0% increase. Correspondingly, the number of vehicles used increases from 33 to 55, while activated vehicle deployment points expand from 7 to 11.

This cost escalation is attributable to the spatial configuration of the network: Station B is located 40–80 km from Station A, whereas at least half of the logistics parks are distributed within 10–40 km of Station A, with the remainder randomly positioned near either station. Consequently, the average transport distance to Station B is generally greater than that to the logistics parks.

When the Station B proportion is low at 30–40%, the system operates efficiently with only 7 deployment points and 33–35 vehicles. A significant cost jump occurs when the proportion reaches 50%, where the objective value increases by 13.9% and an additional deployment point is activated. The cost increase accelerates at higher proportions: from 50% to 60%, costs increase by 24.2% with 2 additional deployment points activated; from 60% to 70%, costs further increase by 10.0%.

### 6.4. Algorithm performance

The sensitivity analysis comprises 15 instances categorized by parameter ranges as follows. Instances 1–5 are related to changes in the Freight Truck Maximum Payload Capacity. Five experimental cases with capacities of 2.5, 3.0, 3.5, 4.0, and 5.0 tonnes.Instances 6–10 are related to changes in the Maximum Allowable Delay Time. Five experimental cases with time limits of 200, 220, 240, 260, and 280 minutes, with each 20-minute extension defining one instance.Instances 11–15 are related to changes in the Cargo Demand Distribution Ratios. Five experimental cases with the proportion of cargo destined for Station B at 30%, 40%, 50%, 60%, and 70%, where each 10% interval defines one instance.

To evaluate the performance of the proposed ALNS algorithm, each of the aforementioned instances was solved using the CPLEX solver, ALNS algorithm, and GA algorithm. The baseline parameters are configured as follows: vehicle capacity C=3.5 tons, maximum allowable time $T_{allow}=240$ minutes, and Station B demand ratio = 50%. When varying one parameter, the other two remain at their baseline values. The comparison results across experimental instances are presented in Table 11.

**Table 11:** Sensitivity analysis results comparison of CPLEX, ALNS, and GA.

Instance (Parameter)	CPLEX			ALNS		GA	
	Status	Cost	Trun(s)	Cost	Trun(s)	Cost	Trun(s)
1 (2.5t)	Feas.	1576826	–	1577417	35.981	1729155	0.587
2 (3.0t)	Feas.	1415763	–	1416123	28.131	1417203	0.689
3 (3.5t)*	Opt.	1260158	7.14	1260158	5.85	1411958	0.46
4 (4.0t)	Opt.	1255926	16.856	1255926	9.785	1255926	0.626
5 (5.0t)	Opt.	1097811	19.977	1097811	4.242	1097811	0.58
6 (200 min)	Feas.	1563041	–	1563041	6.994	1563763	0.697
7 (220 min)	Opt.	1411958	7.957	1411958	4.352	1411958	0.649
8 (240 min)*	Opt.	1260158	7.14	1260158	5.85	1411958	0.46
9 (260 min)	Feas.	1107738	–	1107738	8.946	1108421	0.401
10 (280 min)	Feas.	956713	–	956776	17.722	956713	0.516
11 (30%)	Feas.	1103185	–	1103526	11.99	1104209	0.544
12 (40%)	Feas.	1106099	–	1106441	6.013	1257526	0.654
13 (50%)*	Opt.	1260158	7.14	1260158	5.85	1411958	0.46
14 (60%)	Opt.	1564827	17.426	1564827	15.72	1716697	0.838
15 (70%)	Opt.	1721080	11.036	1721080	4.049	1873080	0.805

The solver CPLEX failed to find optimal solutions within time limit for 7 instances (payload capacities of 2.5t and 3.0t, service time windows of 200 min, 260 min, and 280 min, and demand levels of 30% and 40%), returning only feasible solutions instead. For the remaining 8 instances, it achieved optimal solutions with solving times ranging from approximately 7–20 seconds. These results demonstrate that computational performance varies significantly with parameter settings, reflecting the inherent complexity of the emergency truck dispatching problem under HSR disruptions.

The GA algorithm exhibited the fastest computation times across all instances, consistently solving each case in under 1 second with an average of approximately 0.59 seconds. However, the solution quality showed notable gaps compared to exact methods. For example, in Instance 1 (2.5t), GA produced a cost of 1,729,155—approximately 9.6% higher than CPLEX’s solution of 1,576,826. Similar cost premiums were observed in instances 12 (40%) and 15 (70%), where GA’s costs exceeded the benchmark by 13.6% and 8.8%, respectively.

Across all 15 instances, the proposed ALNS algorithm matched CPLEX’s optimal solutions wherever CPLEX succeeded. Crucially, for the 7 instances where CPLEX failed to achieve optimality, ALNS consistently found solutions comparable to CPLEX’s feasible solutions within seconds with gaps under 0.04%. The algorithm solved every instance in under 36 seconds, with most cases completed in under 12 seconds, significantly outperforming CPLEX in both robustness and efficiency while maintaining superior solution quality compared to GA.

## 7. Conclusions

This study pioneers an emergency dispatching framework for HSR logistics disruptions, integrating deployment point selection and route optimization for the first time. By combining an MILP model with the ALNS algorithm, it achieves the following aspects.

1. Global Optimization under Multi-Constraints. The model unified deployment point selection, timeliness constraints, and multi-route coordination to achieve holistic post-disruption resource scheduling.
2. Dynamic Decision-Making. Designed a dynamic operator strategy combining destroy operators (random/worst/Shaw/depot consolidation removal) and repair operators (greedy/regret-2/regret-3 insertion) to resolve collaborative scheduling challenges.
3. The ALNS algorithm achieves solution quality comparable to CPLEX with a maximum gap of only 0.04% while substantially reducing computation time. While GA offers faster computation, it suffers from significant solution quality degradation. Given the time-critical nature of emergency response to HSR disruptions, rapid solution generation is more valuable than marginal improvements in optimality. ALNS demonstrates significant practical value by achieving near-optimal solutions in seconds rather than minutes, making it well-suited for real-time disruption management where timely decision-making is essential.

This study can provide solutions for HSR logistics interruptions. However, there are several limitations. The current model assumes static demand for goods and does not take into account the dynamic scenarios with concurrent multi-line disruptions. Therefore, in the future, it is possible to extend dynamic demand-responsive models integrating real-time traffic data. In addition, with the increasing emphasis on low-carbon goals, low-carbon scheduling strategies for electric vehicle fleet charging can also be explored, or carbon emission constraints can be introduced.

## Supporting information

S1 File (RAR)

## Abbreviations

HSR: High speed rail
MILP: Mixed-Integer Linear Programming
CRRC: China Railway Rolling Stock Corporation
ALNS: Adaptive Large Neighborhood Search
VRP: vehicle routing problem
EMU: electric multiple units
